# Extensive genomic characterization of a set of near-isogenic lines for heterotic QTL in maize (*Zea mays* L.)

**DOI:** 10.1186/1471-2164-14-61

**Published:** 2013-01-29

**Authors:** Giorgio Pea, Htay Htay Aung, Elisabetta Frascaroli, Pierangelo Landi, Mario Enrico Pè

**Affiliations:** 1 Scuola Superiore Sant’Anna, Piazza Martiri della Libertà 33, 56127, Pisa, Italy; 2 Department of Agroenvironmental Sciences and Technologies (DiSTA), University of Bologna, Bologna, Italy

**Keywords:** Genome-wide genotyping, Single nucleotide polymorphism, Maize, Near-isogenic lines, Heterosis, Linkage drag

## Abstract

**Background:**

Despite the crucial role that heterosis has played in crop improvement, its genetic and molecular bases are still elusive. Several types of structured populations were used to discover the genetic architecture underlying complex phenotypes, and several QTL related to heterosis were detected. However, such analyses generally lacked the statistical power required for the detailed characterization of individual QTL. Currently, QTL introgression into near-isogenic materials is considered the most effective strategy to this end, despite such materials inevitably contain a variable, unknown and undesired proportion of non-isogenic genome.

An introgression program based on residual heterozygous lines allowed us to develop five pairs of maize (*Zea mays* L.) near-isogenic lines (NILs) suitable for the fine characterization of three major heterotic QTL previously detected. Here we describe the results of the detailed genomic characterization of these NILs that we undertook to establish their genotypic structure, to verify the presence of the expected genotypes within target QTL regions, and to determine the extent and location of residual non-isogenic genomic regions.

**Results:**

The SNP genotyping approach allowed us to determine the parent-of-origin allele for 14,937 polymorphic SNPs and to describe in detail the genotypic structure of all NILs. The correct introgression was confirmed for all target QTL in the respective NIL and several non-isogenic regions were detected genome-wide. Possible linkage drag effects associated to the specific introgressed regions were observed. The extent and position of other non-isogenic regions varied among NIL pairs, probably deriving from random segregating sections still present at the separation of lineages within pairs.

**Conclusions:**

The results of this work strongly suggest that the actual isogenicity and the genotypic architecture of near-isogenic materials should be monitored both during the introgression procedure and on the final materials as a paramount requisite for a successful mendelization of target QTL. The information here gathered on the genotypic structure of NILs will be integrated in future experimental programs aimed at the fine mapping and isolation of major heterotic QTL, a crucial step towards the understanding of the molecular bases of heterosis in maize.

## Background

Despite the crucial role that heterosis has played in crop improvement over the years, its genetic and molecular bases are still elusive [1-3]. Several types of structured populations, such as RIL (Recombinant Inbred Line) populations, have been widely used to discover the genetic architecture underlying complex phenotypes, including heterosis. Numerous QTL (Quantitative Trait Loci) for yield and/or yield components related to heterosis were detected in maize [4-6] as well as in several other species [7-18], showing a variety of effects ranging from dominance at various levels to epistasis. However, QTL analysis in structured populations has not been able yet to provide a definite answer on the genetic nature of hybrid vigor. In fact, whereas structured populations are statistically powerful for discovering relevant genomic intervals, they generally lack the statistical power needed for the isolation and the detailed characterization of individual QTL [19]. Accurate estimation of the effects of QTL, including those for heterotic QTL [3], introgressed into near-isogenic materials is considered the most effective strategy for their characterization and the identification of their molecular bases; this in turn could allow incorporating specific QTL into effective breeding programs [20,21]. Near-isogenic Lines (NILs) and Introgression Lines (ILs) [22] are suitable genetic materials to this aim, and can be used as a resource to initiate positional cloning projects and to address more general questions regarding epistatic interactions, genome organization and genetic linkage. General-purpose NIL or IL panels have been developed for QTL analyses in maize, such as those obtained in crosses between B73 and Tx303 [23], between B73 and Gaspe Flint [24] and between B73 and Mo17 [19], with the aim of providing tools for validating QTL and for initiating fine-mapping experiments.

**Figure 1:**
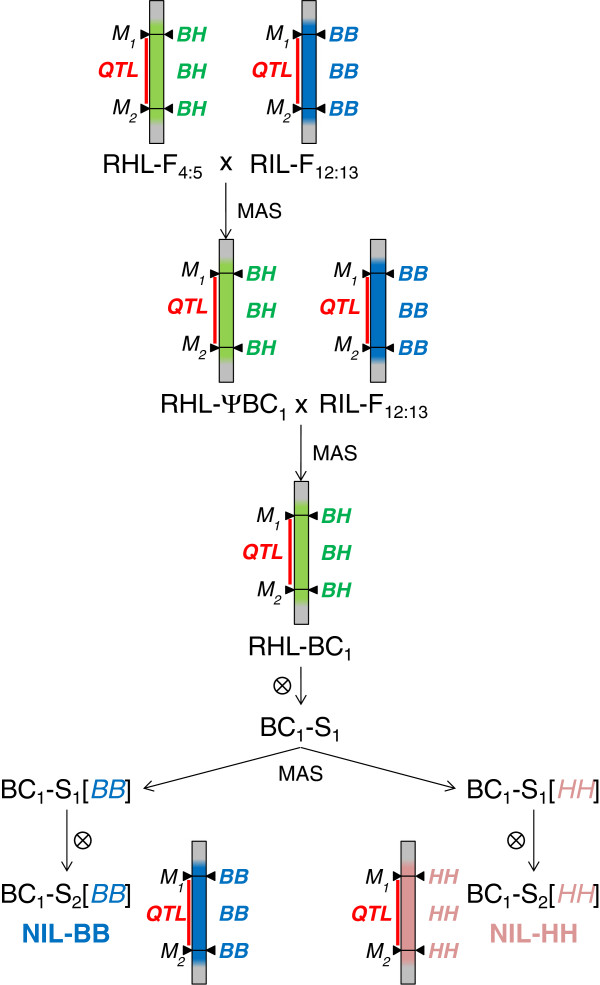
**Introgression scheme adopted for the production of recombinant heterotic NILs.** The two NILs (NIL-BB and NIL-HH) were designed to introgress the target QTL (red bar) in the *B73/B73* and in the *H99/H99* genotype, respectively, while being isogenic anywhere else in the genome. Each pair of NILs was independently obtained starting from different RIL-F_4:5_ individuals (RHL-F_4:5_) selected because heterozygous at the target QTL. These were crossed twice to the corresponding RIL-F_11:13_ as recurrent parent, resulting in the introgression of target QTL into different highly homozygous B73 × H99 recombinant genetic backgrounds. As an example, a back-cross to a hypothetical RIL-F_12:13_ homozygous *B73/B73* at both flanking markers (M_1_ and M_2_) is represented. Application of marker-assisted selection (MAS) is reported alongside arrows which indicate (pseudo)-backcross and selfing generations. Vertical bars represent a chromosome portion of individual plants; blue, pink and green color denote respectively genotypes *B73/B73*, *H99/H99* and *B73/H99*. Genotypes indicated at the QTL are those expected in absence of double recombination events. Abbreviations: RHL, residual heterozygous line; ΨBC_1_, pseudo-backcross one; BC_1_, backcross one; BC_1_-S_1_ and BC_1_-S_2_, first and second selfing generations.

**Figure 2:**
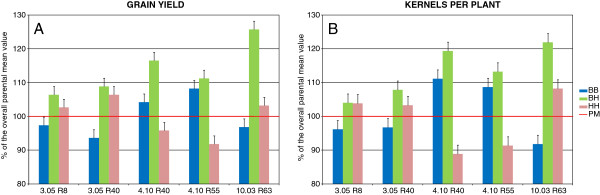
**Performance of heterotic NILs for grain yield and kernels per plant.** Performance, as percentage of the overall parental mean across six field trials (PM, red line), of the three genotypes *B73/B73* (BB, blue), *H99/H99* (HH, pink) and *B73/H99* (BH, green) for grain yield (panel **A**) and for kernels per plant (panel **B**) of QTL 3.05, 4.10 and 10.03 in all five NIL sets. Vertical bars represent the standard error as percentage of PM.

 In previous work undertaken to shed light on the genetic basis of heterosis in maize, we performed a QTL analysis on genetic materials derived from a RIL-F_12:13_ population derived from the single cross B73 × H99 [4]. The level of heterosis underlying genetic effects for several agronomic traits was evaluated, and a number of QTL with heterotic effects on phenotypes were detected. With the aim of identifying the molecular determinants underlying heterotic QTL, we undertook an introgression program based on residual heterozygous lines to develop on-purpose near-isogenic materials [25] suitable for the fine characterization of QTL chosen for their over-dominance effects [4]. In particular, following the crossing-and-selection approach schematized in Figure 1, we obtained pairs of NILs designed to have contrasting genotypes at the selected QTL regions within an otherwise isogenic recombinant genetic background (recombinant NILs). The presence of heterotic phenotypes in these NILs was validated in different genetic combinations and growing conditions [25,26]. In the perspective of fine mapping the introgressed heterotic QTL, we chose to focus our efforts on the five NIL pairs introgressing QTL 3.05, 4.10 and 10.03 in different genetic backgrounds (Table 1); these showed over-dominance for grain yield and number of kernels per plant, which are important agronomic and economic traits. Figure 2 summarizes the performance of these five NIL pairs and their hybrids obtained in our previous studies comprising six field trials with two replications per trial ([26] and unpublished data). Results refer to grain yield per plant and its component number of kernels per plant, the two traits exhibiting the highest level of heterosis. Field performance is expressed as percentage of the parental mean, to allow a comparison among QTL effects. Our data clearly shows that the heterozygote was significantly superior at least to the parental mean in all instances but kernel per plant for 3.05 R8. In two cases, 4.10 R40 and 10.03 R63, the heterozygote was even strongly superior to the best homozygote. Taken together, these data corroborate our previous observations [4,25], justifying the more detailed investigation here presented on these NILs. In fact, although designed as pairs of inbred lines that ideally differ by a single and well defined region, NILs inevitably contain a variable proportion of non-isogenic genome. This is mainly due to linkage drag, that carries segments linked to the one targeted for introgression, and to the presence of other residual unlinked non-isogenic genomic fragments. Therefore, to perform an accurate research, a detailed picture of the genotypic structure of the genetic material under study is paramount. Among the numerous genotyping techniques available, those based on highly parallelized polymorphisms detection are better suited to efficiently tackle this goal. The recent completion of the maize genome reference sequence [27], allowing the physical mapping of SNPs (Single Nucleotide Polymorphisms) and other variations to the maize chromosomes, set the path for the development and testing of highly informative, easy to use and robust genotyping platforms for this species. These platforms are based either upon Comparative Genomic Hybridization (CGH), which also allows the detection of Copy Number Variations (CNVs) relative to the reference genome [28], Genotyping by Sequencing (GBS) methods [29] or large scale SNP genotyping arrays [30]. The latter platform, developed by Illumina, Inc. (San Diego, CA, USA) under the name of MaizeSNP50 BeadChip, is based on SNP markers selected to be preferentially located in genes and evenly distributed across the genome and it has been tested with a large set of maize germplasm, including North American and European inbred lines, parent/hybrid combinations, and distantly related teosinte materials.

**Table 1:** Location and exact length of introgressed QTL regions

**QTL**^**a**^	**Left**	**Position**	**Right**	**Position**	**Interval length (Mbp)**	**Introgression**
	**Marker**^**b**^	**(bp)**	**Marker**^**b**^	**(bp)**		**lines**^**c**^
3.05	*bnlg1505*	147,812,359	*dupssr23*	166,846,373	19.03	RIL 8, RIL 40
4.10	*umc1101*	241,805,620	*umc1109*	243,738,469	1.93	RIL 40, RIL 55
10.03	*bnlg1451*	4,436,646	*umc2016*	62,064,437	57.66	RIL 63

^a^QTL names correspond to the chromosome bins where the QTL were mapped. ^b^Upstream and downstream markers used for marker-assisted introgression. ^c^Introgression lines are the B73 × H99 RIL-F_11:13_ employed as recurrent parents in the introgression scheme (see Figure 1). Positions refer to exact BLASTN local alignment on the B73 RefGen_v1 maize reference sequence (with the correct distance and orientation) of both forward and reverse marker PCR primers (publicly available at MaizeGDB [31]).

Here we describe the results of the detailed genotyping, obtained by using the Illumina MaizeSNP50 BeadChip [30], of five NIL pairs that we specifically produced [25] for the introgression of three heterotic QTL [4]. The objectives of this study were: (1) to assess the reliability of the MaizeSNP50 BeadChip platform and determine its descriptive power on the yet untested B73 × H99 genetic system; (2) to establish the detailed genetic structure of five NIL pairs produced to introgress three heterotic QTL in different recombinant genetic backgrounds; (3) to verify the presence of the expected genotypes within the target QTL introgression regions; (4) to compare genotypic patterns within NIL pairs in order to estimate the extent and position of residual non-isogenic genomic regions.

## Results

### SNP quality control and genomic distribution in the B73 × H99 genetic system

Before proceeding to the genetic characterization of NILs, we assessed the reliability of the MaizeSNP50 BeadChip platform and determined its descriptive power on the yet untested B73 × H99 genetic system. To this aim, we performed thorough quality controls of SNPs and analyzed their genomic distribution based upon the genotypic calls obtained for the NILs’ parental inbred lines (B73 and H99), for their F_1_ hybrid and for two reference B73 samples.

In order to assess the consistency of SNP genotype calls across replicate samples, we compared to each other the two B73 reference samples included as internal controls in the experiment (see Methods for details on the adopted filtering criteria for this specific task). They were identical for all the 47,937 considered SNPs and were subsequently treated as a single sample (indicated as “B73”). B73 sample was then compared to inbred line B73 from Scuola Superiore Sant’Anna (B73-SSA, i.e., the parental line of the original B73 × H99 cross) in order to determine the level of divergence between samples from different seed stocks. The full list of the B73 vs. B73-SSA compared SNP genotypes is available in Additional file 1. A total of 121 and 21 SNPs showed a heterozygous genotype in either B73 or B73-SSA, respectively. The vast majority of SNPs (47,556 SNPs, 99.2%) showed an identical genotype between the two B73 samples, 14 SNPs being heterozygous in both samples. The discordant genotypes at the remaining 381 SNPs (0.79%) were due either to a heterozygous state in one sample but not in the other (142 SNPs) or to discordant homozygous genotypes (239 SNPs). Ninety-five percent (362) of the total discordant SNPs were mapped among chromosomes 2 (110 SNPs, 28.9% of discordant SNPs), 4 (149 SNPs, 39.1%) and 5 (103 SNPs, 27.0%), most of them being within single uninterrupted clusters covering in total about 27 Mbp along intervals 202.7–208.6 Mbp on chromosome 2, 234.6–246.5 Mbp on chromosome 4 and 140.4–149.5 Mbp on chromosome 5.

Before proceeding to the analysis of the genetic structure of NILs, a working subset of 42,771 good quality SNPs (73.9% of the 57,838 SNPs available on the chip) was identified by applying stringent quality filtering criteria which also considered pedigree consistency (see Methods) to the full SNP dataset (genotype calls and quality status of all SNPs is reported in Additional file 2). Briefly, the following number of SNPs were excluded at each subsequent filtering step: 8,319 failed in all samples; 5,386 failed in any samples among B73-SSA, H99 and B73-SSA × H99 F_1_ hybrid; 618 unmapped or mapped to the unknown chromosome; 114 heterozygous in either of the NIL parental lines (of which 29 in B73-SSA, 81 in H99 and 4 in both); 625 having inconsistent genotypes in the B73-SSA × H99 F_1_ hybrid; and, finally, 5 having inconsistent genotype in NILs samples.

**Figure 3:**
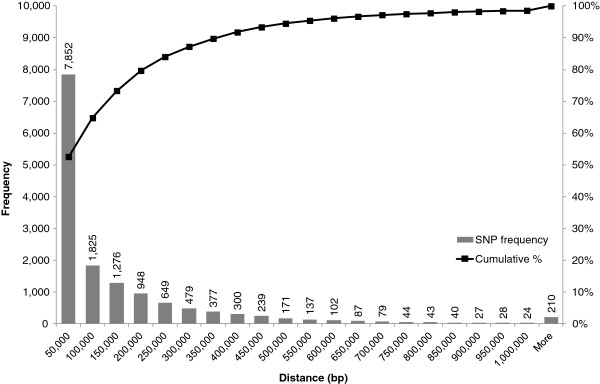
**Genome-wide distribution of distances between adjacent polymorphic SNPs.** Total number of SNPs: 14,937; average distance: 137 kbp; median: 41 kbp; 95th percentile: 525 kbp.

The descriptive power of the MaizeSNP50 BeadChip with respect to the B73 × H99 genetic system was assessed by determining the genomic distribution of the SNP working set among and within the maize chromosomes (Table 2 and Figure 3). The distribution of the 42,771 SNPs (Table 2, “Total”) was found significantly non-uniform among chromosomes due to a significant higher- and lower-than-expected number of SNPs in chromosomes 1 and 4, respectively. The same pattern was also observed when considering all the 55,326 SNPs with known chromosome position originally included in the chip (data not shown), indicating that the overall SNP distribution was not biased by the applied filtering criteria.

**Table 2:** SNPs genomic distribution among chromosomes

**Chromosomes**			**SNP distribution**						
**No.**	**Length (Mbp)**	**%**^**a**^	**Total**		**Polymorphic**		**Monomorphic**		***PM ratio***
			**(T)**		**(P)**		**(M)**		
			**No.**	**%**^**b**^	**No.**	**%**^**c**^	**No.**	**%**^**c**^	
1	300.2	*14.7%*	6,812	*15.9%*▲	2,272	*33.4%*	4,540	*66.6%*▲	**▼ **
2	234.8	*11.5%*	4,910	*11.5%*	1,625	*33.1%*▼	3,285	*66.9%*	**▼ **
3	230.6	*11.3%*	4,697	*11.0%*	1,730	*36.8%*	2,967	*63.2%*▼	**▲ **
4	247.1	*12.1%*	4,770	*11.2%*▼	1,752	*36.7%*	3,018	*63.3%*▼	**▲ **
5	216.9	*10.6%*	4,640	*10.8%*	1,444	*31.1%*▼	3,196	*68.9%*▲	**▼ **
6	169.3	*8.3%*	3,468	*8.1%*	1,238	*35.7%*	2,230	*64.3%*	
7	171.0	*8.4%*	3,539	*8.3%*	1,361	*38.5%*▲	2,178	*61.5%*▼	**▲ **
8	174.5	*8.5%*	3,712	*8.7%*	1,212	*32.7%*	2,500	*67.3%*▲	**▼ **
9	152.4	*7.4%*	3,151	*7.4%*	1,192	*37.8%*▲	1,959	*62.2%*▼	**▲ **
10	149.7	*7.3%*	3,072	*7.2%*	1,111	*36.2%*	1,961	*63.8%*	
**Total**	**2,046**	***100%***	**42,771**	***100%***	**14,937**	***34.9%***^**d**^	**27,834**	***65.1%***^**d**^	

^a^Chromosome length over total genome length; ^b^number of SNPs per chromosome over total number of SNPs; ^c^number of polymorphic (P) and monomorphic (M) SNPs over the total number of SNPs mapped on the respective chromosome; ^d^number of polymorphic (P) and monomorphic (M) SNPs over total number SNPs. Up/down arrows indicate the sign of significant (P < 0.05) deviations from the expected SNP distribution among chromosomes for the corresponding tested null hypotheses, namely: uniform distribution of total SNPs (T); uniform distribution of polymorphic SNPs (P); uniform distribution of monomorphic SNPs (M); independent distribution of polymorphic vs. monomorphic SNPs from their mapping chromosome (*PM ratio*).

The comparison of genotype calls between B73-SSA and H99 showed that 14,937 SNPs, about 35% of the retained SNPs, are polymorphic between the two inbred lines, with proportions ranging from 31.1% in chromosome 5 to 38.5% in chromosome 7 (Table 2, “Polymorphic”). The ratios of polymorphic vs. monomorphic SNPs (Table 2, *PM ratio*) resulted overall non-independent from the chromosome where the SNPs were mapped. Pairwise tests indicated that this was due to significant deviations from independence for all chromosomes except chromosomes 6 and 10. In particular, chromosomes 3, 4, 7 and 9 showed *PM ratios* higher than expected under the hypothesis of independence, whereas the opposite was true for chromosomes 1, 2, 5 and 8. All non-independent *PM ratios* (see Table 2) could be accounted for by various combinations of significant non-uniform distributions among chromosomes of either the polymorphic (P) or the monomorphic (M) SNPs (i.e., the numerator or the denominator of the *PM ratio*, respectively), or both. For example, the significantly low *PM ratio* observed in chromosome 2 corresponds to a significant depletion of polymorphic SNPs only, whereas in chromosome 5 it is due to polymorphic and monomorphic SNPs being at the same time, respectively, less and more than expected by their uniform distribution.

The genome-wide distribution of distances between adjacent polymorphic SNPs is shown in Figure 3. The overall mean distance is 137 kbp, with means per chromosome ranging from 125 kbp in chromosome 7 to 150 kbp in chromosome 5 (data not shown). Half of the polymorphic SNPs fall within 41 kbp from each other and 95% within 525 kbp. Only 210 SNP intervals have sizes exceeding 1 Mbp, 90% of which (187 SNPs) being below 3 Mbp. The maximum SNP interval sizes observed per single chromosome range from 2.9 Mbp in chromosome 3 to 33.1 Mbp in chromosome 5. Noticeably, this latter interval, by far the largest detected (the second one being 8.4 Mbp in size), is mapped between two SNPs at 89.3 and 122.5 Mbp and is centered around the centromere of chromosome 5 (*cent5*, 101.3-108.4 Mbp), the largest annotated maize centromere by a factor of five [31].

**Figure 4:**
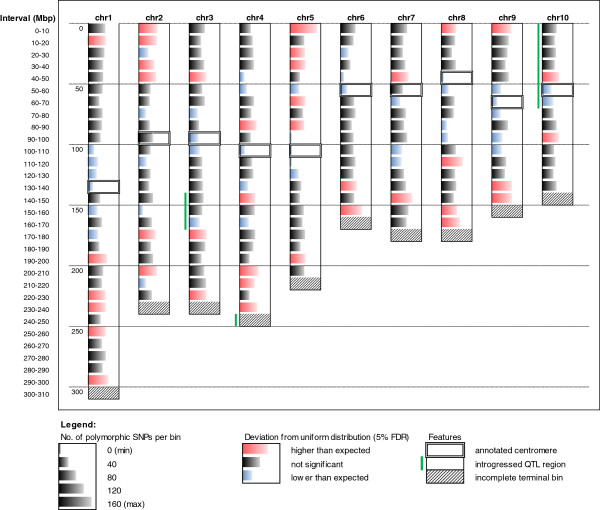
**Distribution of polymorphic SNP within chromosomes.** Bin size: 10 Mbp. Bin colors indicate significant non-uniform distribution of SNPs (see text). Red: over-representation of polymorphic SNPs; Blue: under-representation of polymorphic SNPs. Thick boxes indicate bins containing the annotated centromeres; green vertical bars denote bins containing the introgressed QTL; grey rectangles indicate incomplete terminal bins (excluded from tests).

Polymorphic SNPs were also found significantly non-uniformly distributed within each of the ten maize chromosomes arbitrarily divided in 10 Mbp bins (Figure 4). Orthogonal pairwise chi-square tests corrected for multiple tests with Benjamini-Hochberg False Discovery Rate (FDR < 5%) allowed us to identify significantly low- and high-polymorphic bins within each chromosome, i.e., bins comprising less or more polymorphic SNPs than those expected under the null hypothesis of their uniform distribution within each chromosome. A total of 81 out of the 200 bins (40.5%) were classified as significantly low- (42, blue color) or high-polymorphic (39, red color), the former and the latter being preferentially observed in centromeric and telomeric regions, respectively. In fact, the first five bins located at the two telomeres of each chromosome (i.e. 100 “telomeric” bins, corresponding to half of the represented genome), included 71.8% (28 out of 39) of the high-polymorphic bins and only 15.4% (6 out of 42) of the low-polymorphic ones. Conversely, the 5 bins per chromosome centered on the bin including the annotated centromere (i.e. 50 “centromeric” bins, corresponding to 25% of the represented genome), included 24 out of 42 (61.5%) low-polymorphic bins, against only 4 out of 39 (9.5%) high-polymorphic ones. Noticeably, annotated centromeres map within significantly low-polymorphic bins in all instances except chromosomes 2 and 7. Higher resolution (2 Mbp bins) heat-maps representing the distribution on the maize chromosomes of all good-quality SNPs and of the proportion of polymorphic SNPs within each bin, respectively, are provided in Additional file 3.

### Assessment of allele inheritance patterns in NILs

Five pairs of maize recombinant NILs were analyzed (see Table 1 and Methods for a full description and nomenclature), each designed to introgress one of three heterotic QTL mapping on chromosome bins 3.05, 4.10 and 10.03 [25]. Each pair consists of two inbred lines, named NIL-BB and NIL-HH, designed as to introgress the target QTL in the *B73/B73* and in the *H99/H99* genotype, respectively (Figure 1). Two pairs of NILs were available for QTL3.05 and QTL4.10, whereas only one pair was produced for QTL10.03. The full list of SNP genotype calls and a summary of genotypes distributions among chromosomes of polymorphic SNPs in all NIL samples are reported in Additional file 2 and Additional file 4, respectively. Failure rates in NILs averaged 0.86%, ranging from 0.19% in NIL10.03_R63-BB to 3.2% in NIL4.10_R55-HH, with an observed residual heterozygosity of 0.92% (ranging from 0.04% in NIL3.05_R8-BB to 3.2% in NIL4.10_R55-HH). The lowest and the highest average heterozygosity level per single chromosome were observed in chromosomes 8 (0.18%) and 5 (3.3%), respectively. The genome-wide proportion of B73 homozygous SNPs over all the polymorphic SNPs ranged between 27.7% in NIL4.10_R55-HH and 51.1% in NIL3.05_R8-BB.

**Figure 5:**
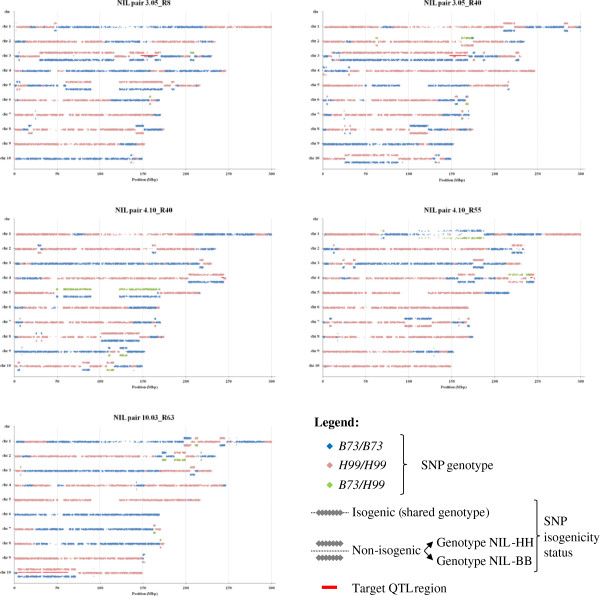
**Genotypic structure and isogenicity of NIL pairs.** Graphs synthesizing information both on the overall genotypic structure of NILs (i.e., position and size of recombination blocks, given by colors) and on genotype and position of non-isogenic regions (unaligned plotting lines). Each panel represent contrasting NILs within a pair. Genotypes at SNPs are color coded (B73/B73 = blue; H99/H99 = pink; B73/H99 = green). For each chromosome, non-isogenic regions are plotted as two separate lines above (SNP score in NIL-HH) and below (SNP score in NIL-BB) a shared line in the middle, where SNPs with the same genotype in both NILs (i.e., isogenic) are plotted. Red bars represent the QTL introgression regions.

Graphical representations of the pattern of allelic inheritance along chromosomes of each NIL (Figure 5) were obtained by plotting color-coded genotypes of all non-failed polymorphic SNP against their positions on the B73 RefGen_v1 maize reference genome [27]. QTL regions 3.05, 4.10 and 10.03 (Table 1) include respectively 122, 40 and 392 polymorphic good-quality SNPs on the maize MaizeSNP50 chip, corresponding in the order to SNP densities of 6.57, 20.69 and 7.15 SNP/Mbp. Analysis of SNP genotypic data indicates that all target QTL regions were introgressed as expected in all the respective NIL pair(s), i.e., as homogeneous regions of homozygous *B73/B73* and *H99/H99* genotypes in NIL-BB and NIL-HH, respectively.

We evaluated also the cross between NIL4.10_R55-BB and NIL4.10_R55-HH, where the heterozygosity level was the highest detected among all NIL materials (834 heterozygous SNPs). Out of the 14,036 polymorphic SNPs successfully scored in all three samples of set 4.10_R55, genotype inheritance in NIL-BH with respect to the genotype observed in its two parental lines (NIL-BB and NIL-HH) could be unambiguously confirmed for 13,858 SNPs (98.7%). Among these, 705 of the 805 (87.6%) heterozygous SNPs overall detected in NIL-BH corresponded to contrasting homozygous genotypes in NIL-BB and NIL-HH. For the remaining 178 non-matching SNPs (1.3%), four different situations were observed: (1) a heterozygous genotype in NIL-BH when one of the parents was heterozygous (96 SNPs in total, 44 heterozygous only in NIL-BB and 52 only in NIL-HH); (2) a heterozygous genotype in all three samples (4 SNPs); (3) a homozygous genotype in NIL-BH and contrasting homozygous genotypes in parental NILs (3 SNPs); (4) a homozygous genotype in NIL-BH when one of the parents was heterozygous (75 SNPs, of which 61 and 14 heterozygous in NIL-BB and NIL-HH only, respectively). Cases (1) and (2) could be accounted for by the co-dominant nature of SNP markers (i.e., heterozygous calls are obtained when genetically heterogeneous samples are scored in bulk), whereas cases (3) and (4) are not expected by segregation. However, the fact that the 3 SNPs of case (3) delimit a region between 157.3 and 176.2 Mbp on chromosome 4 where other 86 adjacent SNPs show consistent genotypes (i.e., homozygous contrasting genotypes in NIL-BB and NIL-HH and a corresponding heterozygous genotype in NIL-BH), suggests that in this case the observed genotypic inconsistency might be actually due to SNP calling inaccuracy in the NIL-BH sample. Besides invoking possible SNP call errors in any of the three samples, a reasonable explanation for the 75 SNPs of case (4) could reside in a “sampling effect”, that is, only NIL-BH individuals homozygous at these SNPs were actually genotyped, having been selected by effect of chance among individuals in fact segregating in a 1:1 proportion.

### Assessment of isogenicity in NILs

A summary of the number, chromosomal distribution and size of all detected non-isogenic regions is reported in Table 3. For the sake of simplicity, the specific chromosome containing the QTL introgressed in each NIL was treated separately from the other chromosomes, and no distinction was made between fully homozygous and partially heterozygous non-isogenic regions. With the exclusion of the specific chromosomes containing the QTL introgressed in each pair, the overall number of distinct non-isogenic blocks per NIL pair fell between 10 (3.05_R8) and 19 (3.05_R40), with an average block length comprised between 3.6 and 13.7 Mbp in NIL pairs 10.03_R63 and 3.05_R8, respectively. The estimated proportion of non-isogenic genome, also excluding the QTL chromosome, ranged from 2.4% in NIL pair 10.03_R63 to 11.5% in pair 3.05_R40. The largest proportions of non-isogenic regions in non-QTL chromosomes were observed for chromosome 5 in NIL pairs 3.05_R8 (54.3%) and 4.10_R40 (55.0%), followed by chromosome 10 in pair 3.05_R40 (44.9%), chromosome 8 in pair 4.10_R40 (26.3%), chromosome 7 in pair 4.10_R55 (20.7%). In three of the remaining cases, non-isogenic regions represented 10 to 20% of each chromosome, whereas in all remaining instances they were below 10%. The average observed non-isogenicity per chromosome, excluding in each case the QTL chromosomes in the corresponding NILs, was 6.8% and ranged from 0.51% in chromosome 4 (*n* = 3) to 22.0% in chromosome 5 (*n* = 5). The largest proportions of non-isogenic regions within QTL specific chromosomes (excluding the QTL region) were observed in both NIL pairs for QTL 3.05, with 63.7% and 46.9% in NIL R40 and R8, respectively. Non-isogenicity for the QTL chromosome were very similar to each other for the two NIL pairs for QTL 4.10, 15.9% and 17.1% in pair R40 and R55 respectively, and much lower than that of the two pairs for QTL 3.05. Finally, the observed residual non-isogenicity in chromosome 10 for the NIL pair 10.03_R63 was 6.0%.

**Table 3:** Summary statistics of the genomic distribution of non-isogenic regions in NIL pairs

**Chromosome**	**Stats**^**a**^	**NIL 3.05**	**NIL 3.05**	**NIL 4.10**	**NIL 4.10**	**NIL 10.03**
		**R8**	**R40**	**R40**	**R55**	**R63**
**1**	***NB***	1	2	-	6	3
	***NS***	15	123	-	183	148
	***TS***	2.5	17.2	-	52.4	17.1
	***CF***	0.84%	5.7%	-	17.5%	5.7%
**2**	***NB***	-	2	2	3	3
	***NS***	-	113	55	96	155
	***TS***	-	16.5	8.1	11.3	17.6
	***CF***	-	7.0%	3.4%	4.8%	7.5%
**3**	***NB***	5^b^	5^b^	1	3	2
	***NS***	762	1,023	52	171	46
	***TS***	108.0	146.8	3.8	15.7	6.3
	***CF***	46.9%	63.7%	1.6%	6.8%	2.7%
**4**	***NB***	-	3	1^b^	3^b^	1
	***NS***	-	35	275	251	2
	***TS***	-	3.8	39.3	42.2	0.00
	***CF***	-	1.5%	15.9%	17.1%	0.0002%
**5**	***NB***	4	1	2	-	-
	***NS***	490	10	459	-	-
	***TS***	117.8	1.2	119.3	-	-
	***CF***	54.3%	0.54%	55.0%	-	-
**6**	***NB***	1	3	-	-	-
	***NS***	25	82	-	-	-
	***TS***	1.4	6.7	-	-	-
	***CF***	0.85%	4.0%	-	-	-
**7**	***NB***	1	3	2	4	1
	***NS***	3	21	22	273	25
	***TS***	0.15	1.8	2.0	35.4	2.3
	***CF***	0.09%	1.0%	1.2%	20.7%	1.3%
**8**	***NB***	2	3	3	1	1
	***NS***	94	225	398	2	16
	***TS***	13.4	22.6	45.9	0.00	1.2
	***CF***	7.7%	13.0%	26.3%	0.00%	0.68%
**9**	***NB***	-	-	3	-	2
	***NS***	-	-	97	-	14
	***TS***	-	-	10.3	-	1.9
	***CF***	-	-	6.8%	-	1.2%
**10**	***NB***	1	2	3	-	1^b^
	***NS***	10	417	144	-	69
	***TS***	1.6	67.2	17.9	-	9.0
	***CF***	1.1%	44.9%	12.0%	-	6.0%
**Total**^c^	***NB***	**10** (15)	**19** (24)	**16** (17)	**17** (20)	**13** (14)
	***NS***	**637** (1,399)	**1,026** (2,049)	**1,227** (1,502)	**725** (976)	**406** (475)
	***TS***	**137.0** (245.0)	**136.9** (283.7)	**207.3** (246.6)	**114.8** (157.0)	**46.3** (55.2)
	***GF***	**7.5%** (12.0%)	**7.5%** (13.9%)	**11.5%** (12.1%)	**6.4%** (7.7%)	**2.4%** (2.7%)

Number, chromosomal distribution and size of detected non-isogenic blocks in each NIL pair are reported. Data referring to the chromosomes containing the introgressed QTL for the corresponding NIL pair are underlined. ^a^Statistics: *NB* = number of non-isogenic blocks; *NS* = number of non-isogenic SNPs; *TS* = total size of non-isogenic blocks (Mbp); *CF* = non-isogenic chromosome fraction; *GF* = non-isogenic genome fraction. B73 RefGen1 genome size: 2,046,341,370 bp. ^b^QTL introgression regions (see Table 1) are excluded from within-chromosome computations; ^c^Totals are reported both excluding and including (in brackets) data from the chromosome containing the introgressed QTL in the corresponding NIL pair.

The chromosome positions of all non-isogenic regions detected for each NIL pair, along with the corresponding SNP genotypes of the individual NILs can be observed in Figure 5. Non-isogenic regions of different extent having concordant genotype with the QTL region were found immediately upstream and downstream of the flanking markers defining each QTL region. The presence of these regions could be ascribed to linkage drag effect specifically associated with the markers employed for the introgression of each QTL. Non-isogenicity around QTL 3.05 extended asymmetrically by 17.3 Mbp in NIL pair 3.05_R8 (13.4 Mbp upstream and 3.9 Mbp downstream) and by 145.2 Mbp in NIL pair 3.05_R40 (128.1 Mbp upstream and 17.1 Mbp downstream). In the former pair, an additional non-isogenic region of 86.7 Mbp detected further upstream to the QTL region largely overlapped with the more extended non-isogenic region detected in NIL pair 3.05_R40. A comparable linkage drag effect observed in the two genetic backgrounds suggests that it might be specifically associated with the markers employed for introgressing the QTL. In NIL pair 3.05_R40, two additional adjacent non-isogenic regions harboring opposite homozygous genotypes, being probably the effect of two close recombination events around positions 19.2–19.6 Mbp and 12.2 Mbp, were also detected immediately preceding the upstream non-isogenic extended region, expanding it of additional 9.4 Mbp (up to position 10,302,495 bp). In this same NIL pair, a small sub-segment within the QTL region (19 adjacent SNPs between positions 152.0 and 154.4 Mbp) resulted isogenic, and homozygous *H99/H99*, in the two contrasting NILs. Non-isogenicity around QTL 4.10 also extended beyond the QTL boundaries in both NIL pairs, also suggesting the presence of linkage drag, although of a more limited extent. In this case, non-isogenic regions extended by about 39.3 Mbp (39.0 Mbp upstream and 0.3 Mbp downstream) in NIL pair R40 and by about 6.9 Mbp (4.9 Mbp upstream and 2.0 Mbp downstream) in NIL pair R55. Albeit non contiguous, the non-isogenic region in pair 4.10_R55 might be considered to extended upstream for further 20.8 Mbp (up to position 216.1 Mbp) for a total of about 25.7 Mbp upstream, thus overlapping to a larger extent that of the other QTL 4.10 NIL pair, similarly to what observed above for the QTL 3.05 NIL pairs. Finally, the non-isogenic regions flanking QTL 10.03 region extended about 9.3 Mbp (0.2 Mbp upstream and 9.1 Mbp downstream).

Additional unlinked non-isogenic regions between contrasting NILs were also detected in all pairs and mapped genome-wide. Non-isogenic blocks were generally found as stretches of SNPs of coherent genotypes, whereas recombination within non-isogenic blocks was observed in a few cases only (e.g. blocks on chromosomes 3 and 6 in NIL pair 3.05_R40). Chromosomal regions having a heterozygous genotype in one of the compared NILs only constitute for the most part full independent blocks, being only in fewer instances one of the two boundaries of otherwise larger homozygous non-isogenic blocks. The only exception to this latter situation was observed in NIL pair 3.05_R40 for the non-isogenic block mapped at interval 130.5-135.7 Mbp on chromosome 10 (20 SNPs), which consists in fact of three sub-blocks, the middle one of which being heterozygous in NIL-BB only. Only 2–3 isolated SNPs per NIL pair are heterozygous in both NILs. Finally, positions of unlinked non-isogenic blocks across all NIL pairs appear to be largely unrelated. A more extended overlapping of non-isogenic blocks could be observed in NIL pairs 3.05_R40 and 4.10_R40, clearly a manifestation of their relatedness since they were developed in the same genetic RIL 40 background.

## Discussion

In this study we undertook the analysis of the detailed genotypic structure of near-isogenic materials specifically produced for the introgression of three heterotic QTL in maize that we detected and then further characterized [4,25,26]. The NIL pairs hereby analyzed represent unique material for the study of hybrid vigor in maize, since the analysis of mendelized heterotic QTL might shed light on some relevant, and possibly general, genetic and molecular mechanisms underlying this phenomenon. Given this premise, it is paramount that great efforts are made to acquire a detailed knowledge of the genetic structure of such materials, possibly on a whole-genome scale. In fact, accurate determination of the actual isogenicity of NILs at a genome-wide level must not be neglected, since conclusions derived on the effects of QTL mendelizing in NIL materials largely rely upon it. Pursuing this goal, we genotyped the original inbred lines (B73-SSA and H99) and the recombinant NILs obtained from their cross by the Illumina MaizeSNP50 BeadChip. This platform allows the scoring of predetermined genotype variants at more than 50,000 SNPs selected upon a large maize diversity panel and was recently tested on several US inbred lines [30]. The Illumina MaizeSNP50 BeadChip system was chosen because, in the context of a bi-parental genetic system based on common US inbred lines such as the one under analysis, it provided the best combination of potential information content, technical reliability, and resolution needed to ascertain the overall genetic structure and the level of isogenicity of NILs. It has been proposed that heterosis might be associated to large structural variations in the genome leading to complex patterns of gene complementation through the combination of the dispensable genomes within the extremely diverse maize germplasm [32]. The platform chosen for the present analysis, differently from others based upon CGH or re-sequencing techniques, is not suited for addressing the study of such variations, which were at this point beyond the scope of the present work, even though they might indubitably be relevant for a closer investigation on the molecular nature of the introgressed QTL.

First of all we compared the genotypic structure of B73-SSA, the inbred line from which all NILs were derived, with that of the reference B73 accession, for which both replicate SNP scoring produced identical results, confirming the technical reproducibility of genotype calls. The few differences detected between B73-SSA and B73 were in line with the level of inconsistency already observed with duplicate samples from different seed sources [30]. Overall, B73-SSA resulted less heterozygous than its reference counterpart, which might reflect an actual higher homozygosity for this accession. A sampling effect cannot be discarded, since the DNA of B73-SSA was obtained from a small pool of 5–6 seedlings. Quality filtering criteria on SNPs produced 42,771 good quality SNPs vs. the 49,585 SNPs retained in a previous study including 274 maize samples [30] where, as in the present study, a control upon pedigree consistency was made on parent/offspring triplets including the F_1_ hybrid and its parental lines. In our case, this procedure had the additional purpose, due to the lack of replicate samples, to reduce the chances of spurious polymorphism detection and consequent inaccurate genotype calls in NILs samples. It must be considered that the quality of the genotyping data was assessed by Ganal and coworkers with respect to an average failure rate based on a large sample set [30], rather than on a single comparison as in the present study. Despite this difference, however, when removing the 8,628 SNPs having a failure rate > 5% from the good quality SNPs reported by Ganal and coworkers, the resulting number of SNPs (40,957) is strikingly similar to that of SNPs never failed in any sample in the present study (40,852), confirming the reliability of the genotyping platform. The application of stringent quality criteria inevitably caused a reduction of the number of SNPs available to the analysis, with the obvious advantage of providing, on the other hand, a more reliable genotype data set. An indication of this aspect came from the fact that the last filtering step (i.e., the removal of inconsistent SNPs present in any NIL sample) caused the exclusion of 5 additional SNPs only. SNPs heterozygous in either of the parental lines were filtered out upon considering both their reduced number, thus their marginal effect on the overall picture, and the fact that their inheritance by descent to the offspring could not be used to unambiguously determine NILs’ genotypic structure at the respective loci. No inferences on the presence of null alleles were made upon failed SNP calls, in order to avoid both an undesirable increase in the genotyping error rate and the use of dominant-type data which do not allow scoring heterozygosity. The maintenance of the distribution pattern of mapped SNPs after quality filtering further indicated that the informative content of the chip, although inevitably reduced, was not overall biased by the filtering process.

The number of polymorphic SNPs detected between B73-SSA and H99 inbred lines by the Illumina MaizeSNP50 chip was adequate for the purposes of the present study, which was to describe the detailed genetic structure and genotype inheritance patterns in bi-parental materials from them derived. However, no absolute considerations upon the polymorphism level hereby detected between these two lines could be made, nor any comparisons with those previously detected among others. In fact, despite being designed upon a large maize germplasm panel, many of the SNP markers present on the Illumina MaizeSNP50 chip were selected upon data available from inbred lines B73 and Mo17. An anomalous high number of polymorphic SNPs (ca. 52%) with respect to previous knowledge about genetic diversity in maize was observed between these two lines when analyzed by this SNP platform in the original study on a large maize diversity panel [30]. This suggested the presence of an ascertainment bias associated to the design of this SNP chip, which has been further confirmed more recently by a diversity analysis extended to a panel of 77 elite European inbred lines [33]. In the context of germplasm organization, inbreds are commonly assigned to heterotic pools according to estimates of their genetic similarity [34]. Surprisingly, however, inbred line H99, analyzed in the present work for the first time with this platform, resulted more similar to B73 than to Mo17 (respectively 35% vs. 41% of polymorphic SNPs, calculated on the same set of 42,771 good quality SNPs; Pea et al., unpublished data), despite the fact that H99 and Mo17 both belong to the Lancaster Sure Crop heterotic group [35].

Our analysis showed that the distribution of SNPs in the genome is in general not uniform. Considering in particular the chromosomes bearing the introgressed QTL, chromosomes 3 and 4 showed a high polymorphic vs. monomorphic ratio, in both cases due to a lower than expected number of non-informative monomorphic SNPs. Chromosome 4 has also a significant low number of SNPs when compared to other chromosomes. However, SNP density varies sensibly within each chromosome, showing a marked tendency for an over-representation of polymorphic SNPs in telomeric regions which has been already observed for IBM and LHRF populations [30]. This aspect might reflect the constraint in the SNP discovery process towards the use of unique, and thus genic, sequences, which tend to be more abundant in telomeric regions. This biased distribution of SNPs also affected the density of SNPs within the different introgressed QTL regions. In fact, the number of total SNPs per Mbp is 20.12 for QTL 3.05 and 17.34 for QTL 10.03, and more than twice as much (42.42) for QTL 4.10 region, which maps at the telomere of long arm of chromosome 4. This relative difference is even larger when considering polymorphic SNPs only, which are 6.57 and 7.15 per Mbp for QTL 3.05 and QTL 10.03, respectively, against 20.69 per Mbp in QTL 4.10 region.

The distances between adjacent polymorphic SNPs between B73 and H99 present on the chip, being for the vast majority shorter than 1 Mbp, allowed us to draw detailed maps of the genetic structure of this unique NIL material, to our knowledge the only available introgression material for heterotic QTL in maize. First of all, we obtained an accurate definition of the allelic structure at QTL regions, where the successful introgression of coherent chromosome blocks of the expected genotypes within the flanking markers used for marker-assisted selection (MAS) was confirmed in all contrasting NILs. Therefore, polymorphic SNPs mapping within the QTL introgression regions represent high-density markers that can be used for the fine mapping of the underlying heterotic QTL through the scoring of QTL-specific segregating populations derived from the cross of contrasting lines within each of the NIL pairs. Recombination events and regions of non-isogenicity were also pinpointed genome-wide for all NIL pairs to an unprecedented level of detail, also establishing an invaluable asset towards the characterization and the isolation of the introgressed QTL. In particular, the homogeneous introgression of the same QTL in NIL pairs having different, and known, recombinant structures will allow us to undertake a fine mapping approach of QTL 3.05 and QTL 4.10 in distinct, yet comparable, near-isogenic segregating populations. Such an approach might allow, in turn, the detection of epistatic effects and the isolation of disturbing factors, thus increasing the chances of both characterizing and fine mapping these QTL. In the case of NIL pairs 3.05_R8 and 3.05_R40, the target QTL region is interrupted by two and one isogenic regions, respectively. However, experimental evidences show that the QTL effect is still present in both NIL pairs [25,26], suggesting that in both cases the QTL might map within the spared non-isogenic sub-regions, which in turn might represent *per se* a refinement of QTL 3.05 mapping position.

In all NILs SNPs were found organized along chromosomes in blocks of different length bearing concordant genotypes, consistent with the presence of coherent bi-parental chromosomal recombination blocks, as expected given the adopted introgression design [25], and further supporting the reliability of the adopted SNPs genotyping platform. The assessment of the allelic inheritance patterns at the genome wide-level allowed us to identify unforeseen non-isogenic regions present outside the target QTL regions in contrasting NILs. These regions generally consisted of clusters of adjacent SNPs having coherent contrasting genotypes, rather than being made of isolated discordant SNPs. Non-isogenic regions were found immediately flanking both sides of all target QTL, largely due to the effects of linkage drag associated with the specific markers used for MAS. These effects appear to be specific to the markers used for introgression, since non-isogenic regions of comparable size were observed flanking the same QTL independently introgressed in different RIL backgrounds (i.e., QTL 3.05 in RILs 8 and 40 and QTL 4.10 in RILs 40 and 55). The fact that the effects of linkage drag might be correlated to variable recombination rates along chromosomes is supported by data produced using the same genotyping platform in two maize recombinant populations [30]. The large linkage drag observed on the centromeric side of QTL 3.05 in both NIL pairs corresponds to a chromosomal region of low recombination rate as compared to the region on the telomeric side of the QTL. This latter region in fact is characterized by a much more limited linkage drag in both NIL pairs introgressing QTL 3.05. The higher recombination rate associated to the telomere of chromosome 4 long arm might instead account for the more limited extension of linkage drag associated to QTL 4.10. Finally, a region of exceptionally low recombination rate immediately surrounded by areas of high recombination rate roughly corresponds to the introgression region of QTL 10.03. The former characteristic (low recombination) would explain the consistency of genotypes observed for the contrasting NILs along the whole length of this extended introgression region, whereas the latter (high recombination) might account for the very limited linkage drag observed for this region.

The non-isogenic regions detected in other parts of the genome are randomly distributed and, from the comparison between NIL pairs introgressing the same QTL in distinct backgrounds, do not appear to be related to the QTL introgression procedure. They rather seem to largely reflect the presence of different residual heterozygous regions peculiar to the single BC_1_-S_1_ individuals from which the progenitors of the contrasting NILs in each pair were selected, although other random effects that possibly occurred throughout the inbreeding and selection scheme adopted for the QTL introgression cannot be excluded. The remarkable genotypic similarity observed between NIL pairs having a common ancestor (i.e., 3.05_R40 and 4.10_R40) clearly shows that sizes and distribution of recombination blocks reflect the genetic structure of the specific RIL genotype originating each NIL pair. However, despite the fact that NILs were produced through the same breeding scheme, different level of non-isogenicity were observed in different pairs. This can be ascribed to the effective residual heterozygosity of the single progenitor F_4:5_ sister plants employed for the independent introgression procedures that led to the production of each NIL pair [25]. The average proportion of non-shared alleles over all the polymorphic SNPs (excluding the QTL introgression region, which were influenced by MAS) should roughly coincide with the fraction of the genome, also excluding the QTL target region, expected to be heterozygous when crossing contrasting NILs within a pair. This was in fact what we observed for NIL pair 4.10_R55, where the proportion of non-shared alleles between the contrasting NILs was 6.0% against an observed residual heterozygosity of 5.1% in their hybrid (NIL4.10_R55-BH).

## Conclusions

The adopted SNP genotyping strategy allowed us to draw detailed maps of the genetic structure of unique NIL materials introgressing agronomically relevant heterotic QTL, to establish the successful introgression of the expected genotypes in target QTL regions in all NILs pairs and to detect residual regions of non-isogenicity genome-wide. Effects of linkage drag were found to be related to some extent to the particular region of introgression, whereas other residual non-isogenic regions appeared not to be related to the marker-assisted introgression procedure. The results of this work clearly show that the level of isogenicity and the genetic architecture of near-isogenic materials cannot, and must not, be taken for granted on the sole assumption of theoretical expectations. On the contrary, a detailed molecular characterization of NIL materials, possibly during the introgression process and surely on the final products, is a paramount pre-requisite for a successful mendelization and, eventually, isolation of target QTL.

The extensive characterization of the NIL materials here presented constitutes an invaluable asset towards the characterization and the isolation of the introgressed heterotic QTL. In fact, the integration of the high-density SNP markers identified within the QTL introgression regions and of the genomic information gathered on the architecture of these NILs will greatly improve the scope of future experimental programs aimed at the fine mapping and isolation of major heterotic QTL, a crucial step towards the understanding of the molecular bases of heterosis in maize.

## Methods

### Plant materials

Five pairs of maize NILs were analyzed, consisting of BC_1_-S_2:5_ lines derived by single-seed descent from BC_1_-S_2_ lines previously developed [25]. The two NILs in each pair, named NIL-BB and NIL-HH, were designed to introgress the target QTL in the *B73/B73* and in the *H99/H99* genotype, respectively, while being isogenic anywhere else in the genome. Each pair of NILs was independently obtained starting from different RIL-F_4:5_ individuals selected because heterozygous at the target QTL (RHL-F_4:5_) and then crossed twice to the corresponding RIL-F_11:13_ as recurrent parents, according to the scheme reported in Figure 1. This resulted in the introgression of target QTL into different highly homozygous B73 × H99 recombinant genetic backgrounds [25]. NIL pairs here analyzed introgress each one of three heterotic QTL (designated here as QTL3.05, QTL4.10 and QTL10.03) mapping, respectively, on chromosome bins 3.05, 4.10 and 10.03. More precisely, QTL regions are defined here as the regions on the maize reference sequence comprised between the two public SSR (Simple Sequence Repeats) markers employed as left and right flanking markers in the marker-assisted introgression process (Table 1). Two pairs of NILs each were produced for QTL3.05 (NIL3.05_R8 and NIL3.05_R40, respectively in RIL-F_11:13_ backgrounds no. 8 and 40) and QTL4.10 (NIL4.10_R40 and NIL4.10_R55, respectively in RIL-F_11:13_ backgrounds no. 40 and 55), whereas only one pair was produced for QTL10.03 (NIL10.03_R63, RIL-F_11:13_ background no. 63).

Parental inbred lines B73 and H99, belonging respectively to Iowa Stiff Stalk Synthetic (BSSS) population (Reid Yellow Dent heterotic group) and to Illinois Synthetic 60C population (Lancaster Sure Crop heterotic group) [35], plus their B73 × H99 F_1_ hybrid were included in this study as reference samples for parent-of-origin allele assignment and pedigree consistency check on the genotype calls. As a further control for pedigree consistency in NIL pairs, the NIL hybrid obtained by crossing the contrasting NILs within pair 4.10_R55 (i.e., NIL4.10_R55-BH = NIL4.10_R55-BB × NIL4.10_R55-HH) was also included in the present study. Seed stocks of the two parental inbred lines were the same originally used for the development of all the genetic materials derived from the B73 × H99 RIL-F_11:13_ population [36,37] employed both for QTL mapping [4] and for developing the hereby analyzed NIL pairs [25]. In particular, the B73 parental inbred line will be denoted here as “B73-SSA” (Scuola Superiore Sant’Anna) as to distinguish it from the reference B73 inbred line also included in this study as a reference control.

### High-throughput SNP genotyping and quality check

For each sample, DNA was extracted from 100 mg of young leaf tissues pooled from six seedlings by GenElute™ Plant Genomic DNA Miniprep Kit (Sigma-Aldrich, St. Louis, MO) as per the manufacturer’s protocol (with a single final elution in 100 μl TE 1X). Genomic DNA was checked for quality by electrophoresis on 1% agarose gel and quantified by absorbance using the Nanodrop™ 2000 spectrophotometer (Thermo Fisher Scientific Inc., Waltham, MA). A total of 14 genomic DNA samples (1.5 μg each, lyophilized) were sent at R.T. to TraitGenetics GmbH (Gatersleben, Germany) to be processed on the MaizeSNP50 BeadChip (Illumina, Inc. San Diego, CA, USA). Two independent genomic DNA stocks of the reference B73 inbred line were included by TraitGenetics GmbH as genotype reference controls in the experiment. Allele calling was performed by TraitGenetics GmbH through full automatic processing of raw data using the latest version of the cluster file officially released for this platform (MaizeSNP50_B; M. Ganal, personal communication) and SNP genotype calls were provided as tables in MS Excel format. No inferences on possible insertion/deletion (INDEL) polymorphisms (null alleles) were made upon failed SNP calls, since the employed genotyping technology is not adequate to discriminate such occurrences from experimental failures. SNP mapping positions refer to their chromosome coordinates on the reference B73 maize genome assembly (RefGen_v1) as provided by TraitGenetics GmbH as MS Excel along with genotype calls. When comparing B73-SSA vs. the two reference B73 samples, all SNPs with unknown mapping position (or mapping to the unknown chromosome) and SNPs with failed calls either in B73-SSA or in both reference B73 samples were discarded. For SNPs with failed calls only in one of the reference B73 samples, the genotypes were assigned on the base of the remaining reference call. Before proceeding to the analysis of NIL data, SNPs that matched any of the following criteria (applied in the indicated order to the original full list) were removed from the dataset (quality filtering): (1) failed calls in all samples; (2) failed calls in one or more samples among B73-SSA, H99 or B73 × H99 F_1_ hybrid; (3) unknown mapping position or mapping to the unknown chromosome; (4) heterozygous calls in B73-SSA, H99 or both; (5) inconsistent genotype in the B73 × H99 F_1_ hybrid (i.e., SNPs polymorphic between B73-SSA and H99, but not showing a heterozygous genotype in their F_1_ hybrid); (6) inconsistent genotype in NILs (i.e., SNPs monomorphic between B73-SSA and H99, but showing the alternative allele in any NIL sample).

### Analysis of the genomic distribution of SNPs

All mapped, good-quality, monomorphic and polymorphic classes of SNPs were individually tested for uniform distribution among chromosomes (with respect to chromosome length in bp) by goodness-of-fit chi-square tests with the appropriate degrees of freedom (*df*). Polymorphic SNPs were also tested for uniform distribution within single chromosomes after assigning them to 10 Mbp bins according to their chromosome position. In this case, the expected number of SNPs for each bin was calculated on a per chromosome basis, applying Benjamini-Hochberg FDR method for multiple test correction to significance levels [38]. SNPs that mapped to the last, and thus incomplete, bin of each chromosome were omitted from these intra-chromosome distribution tests. Finally, the independent distribution of polymorphic vs. monomorphic SNPs among chromosomes was assessed by a chi-square test for independence. Whenever applicable, *df*’s available from the above described general tests were decomposed in order to perform (*df* – 1) orthogonal tests aimed at identifying the classes (i.e., chromosomes or chromosome bins) contributing with significant deviations to the rejection of the null hypothesis in the respective general tests.

### Assessment of allelic inheritance patterns of NILs and identification of non-isogenic regions

Parent-of-origin alleles were determined for each SNP that resulted polymorphic between the parental inbred lines (B73-SSA and H99). SNPs inheritance patterns in NILs were then obtained by plotting the genotypes of polymorphic SNPs against their chromosome positions. The direct comparison of the genotypes of polymorphic SNPs between NILs within each pair allowed us to identify non-isogenic regions both within and outside the target QTL introgressed segments. Plots representing both the genotype and the isogenicity status of all polymorphic SNPs against their chromosome positions were obtained in MS Excel for each NIL pair in order to visualize at once both the genotypic structure of each NIL and all the detected non-isogenic regions between contrasting NILs within each pair.

## Abbreviations

B73-SSA: B73 Scuola Superiore Sant’Anna; CGH: Comparative Genomic Hybridization; CNV: Copy Number Variation; df: Degrees of freedom; FDR: False Discovery Rate; GBS: Genotype by Sequencing; IL: Introgression Lines; MAS: Marker-assisted selection; NIL: Near-Isogenic Lines; QTL: Quantitative Trait Loci; RIL: Recombinant Inbred Lines; SNP: Single Nucleotide Polymorphism; SSR: Simple Sequence Repeats.

## Competing interests

The Authors declare that they have no competing interests.

## Authors’ contributions

GP prepared the molecular samples, analyzed the data and drafted the manuscript. GP, EF, PL and MEP produced the NIL materials, conceived and designed the study. HHA and EF participated in analyzing the data. GP, HHA, EF, PL and MEP interpreted the data and critically revised the manuscript. All authors read and approved the final manuscript.

## Authors’ information

GP current address: CeRSA - Plant Genomics Group, Parco Tecnologico Padano, Via Einstein - Loc. Cascina Codazza, 26900 Lodi, Italy.

## Supplementary Material

Additional file 1 **Comparison of SNP genotypes in B73-SSA vs. B73 reference samples.** This table reports the genotypes of B73-SSA and of the two reference B73 samples at all SNPs available on the MaizeSNP50 chip. Click here for file

Additional file 2 **List of all SNPs with quality and polymorphism status descriptors.** This table reports the genotypes of all experimental samples analyzed at all SNPs available on the MaizeSNP50 chip. The last two columns report, respectively, the quality status of all SNPs and the monomorphic/polymorphic status of good quality SNPs. Click here for file

Additional file 3 **Heat maps of SNP genomic distribution.** Genomic heat maps (2 Mbp bins) of the number of all mapped good-quality SNPs and of the proportion of polymorphic SNPs (A3 page size). Click here for file

Additional file 4 **Statistics on polymorphic SNP genotyping scores in NILs.** These tables report the chromosomal distribution of polymorphic SNPs genotypes in each NIL pair. The bottom table (“Total”) reports the average distribution of genotypes across all NIL samples (excluding NIL 4.10_R55-BH). Click here for file
