# Supplementary material for: Extensive genomic characterization of a set of near-isogenic lines for heterotic QTL in maize (Zea mays L.)
Source: BMC Genomics. 2013 Jan 29;14:61. doi: 10.1186/1471-2164-14-61 (PMC3567934; doi:10.1186/1471-2164-14-61)

Genomic distribution of all good quality SNPs

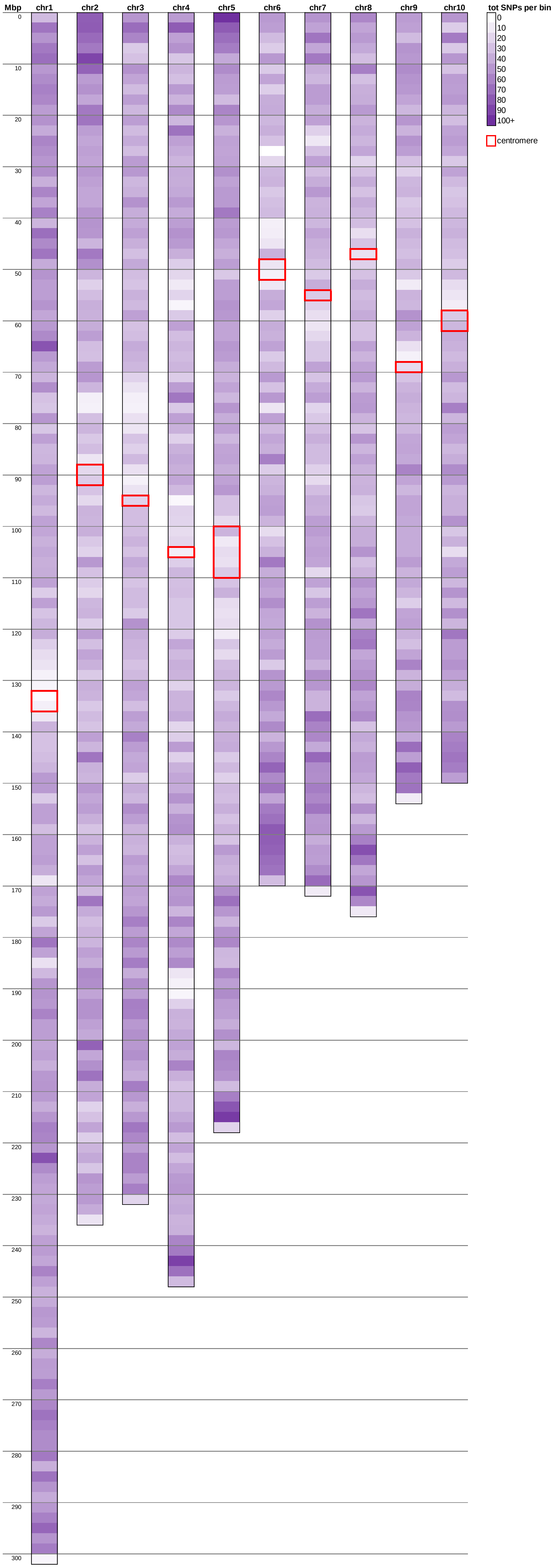

Genomic distribution of polymorphic SNPs (proportions)

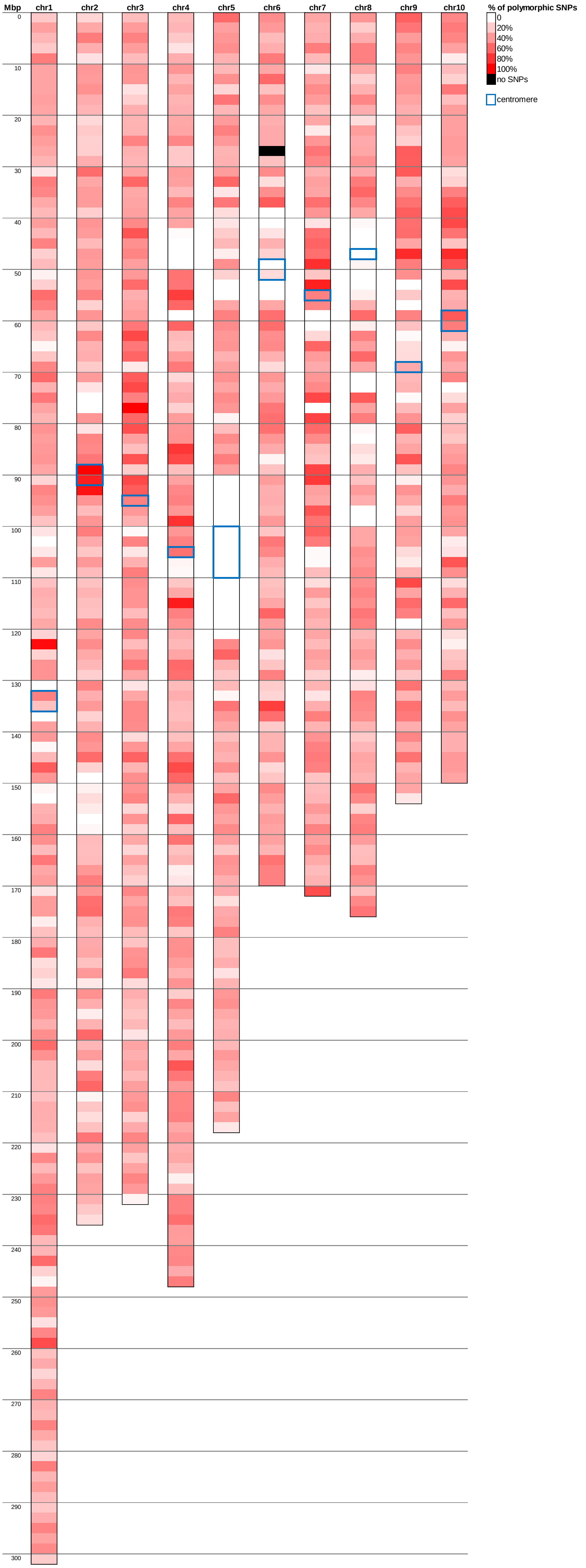

Supplement: Additional file 3 — Heat maps of SNP genomic distribution. Genomic heat maps (2 Mbp bins) of the number of all mapped good-quality SNPs and of the proportion of polymorphic SNPs (A3 page size). [file 1471-2164-14-61-S3.pdf]
